# Risk Profile for Eating Behaviours and Body Perception in a Convenience Sample of Adults: Implications for Health Policy and Person-Centred Care in Primary Healthcare

**DOI:** 10.3390/healthcare14193203

**Published:** 2026-09-28

**Authors:** José Manuel Hernández Garre, Irene Martínez Martínez, Rosa María Cardaba García, Verónica Velasco González, María Inmaculada Pérez Soto, Lucía Pérez Pérez, María José Ferreira Díaz, Isabel Morales Moreno, María Ángeles Navarro Perán, Baldomero de Maya Sánchez

**Affiliations:** 1 Department of Health Sciences, Social Anthropology Area, Catholic University of Murcia (UCAM), 30107 Murcia, Spain; jmhernandez@ucam.edu (J.M.H.G.);; 2 Department of Nursing, University of Murcia, 30120 Murcia, Spain; 3 Department of Nursing, University of Valladolid, 47003 Valladolid, Spain; 4 University School of Nursing, University of Santiago de Compostela, 15782 Santiago de Compostela, Spain

**Keywords:** person-centred care, eating disorders, body image perception, primary care, health policy, risk profile, digital health

## Abstract

**Highlights:**

**What are the main findings?**

- A clear and specific sociodemographic risk profile is identified within the general population, concentrating primarily on young female smokers.
- Multivariate analysis demonstrates that this profile interacts with key behavioural and digital factors, such as an active lifestyle, excessive social media usage, and eating in isolation.

**What are the implications of the main findings?**

- These findings allow for the precise stratification of the target population to optimise preventive interventions in primary care settings.
- An imperative need is evidenced to redesign person-centred clinical protocols by integrating digital literacy and specific screening tools.

**Abstract:**

**Background/Objectives:** Person-centred care necessitates addressing the growing biopsychosocial vulnerability within public health. This study analysed eating behaviours and sociocultural pressures regarding physical appearance in a convenience sample of adults to help inform potential evidence-based policies and programmes in primary care tailored to these exploratory symptom correlates. **Methods:** A quantitative, observational, cross-sectional study was conducted with 1287 participants. Data were collected via an online platform using the Eating Attitudes Test (EAT-26) and the Sociocultural Attitudes Towards Appearance Questionnaire (SATAQ-4). Descriptive statistics, bivariate tests, multiple linear regression models, and complementary binary logistic regression models were utilised to identify variables independently associated with scores and clinical risk thresholds (EAT-26 ≥ 20). **Results:** Multiple linear regression analysis determined that the scores indicating potential risk of altered eating behaviours (EAT-26) was primarily associated with female sex (β = 0.134, *p* < 0.001), followed by never eating in company (β = 0.149, *p* < 0.001), an active lifestyle (β = 0.094, *p* = 0.001), excessive social media usage (β = 0.088, *p* = 0.002), and smoking (β = 0.091, *p* = 0.001). Likewise, susceptibility to sociocultural pressures (SATAQ-4) showed a major association with excessive digital connectivity (β = 0.159, *p* < 0.001), female sex (β = 0.094, *p* = 0.001), smoking (β = 0.078, *p* = 0.004), both sometimes and never eating in company (β = 0.078, *p* = 0.008 and β = 0.082, *p* = 0.005 respectively), and younger age (β = −0.202, *p* < 0.001). Furthermore, the binary logistic sensitivity analysis confirmed that a high clinical risk score (≥20) was independently associated with female sex (Exp(B) = 2.660), smoking (Exp(B) = 2.381), never eating alone (Exp(B) = 2.253), excessive social media use (Exp(B) = 1.665), being active (Exp(B) = 1.500), a greater number of children (Exp(B) = 1.402), and younger age (Exp(B) = 0.969). **Conclusions:** Due to the non-probabilistic convenience nature of the sample, these findings cannot be directly generalized to the general population; however, they delineate the vulnerable demographic profile—young, active, female smokers who are frequent social media users and habitually eat alone—which may help generate hypotheses for future primary care prevention strategies. Furthermore, these results underscore the potential value of integrating specific screening considerations and digital literacy into person-centred healthcare, pending longitudinal validation.

## 1. Introduction

Person-centred care constitutes both an ethical imperative and a fundamental driver of innovation in contemporary healthcare systems [1]. Within primary care and nursing practice, delivering truly individualised, holistic and preventive care requires the design of novel clinical strategies, transformative educational approaches, and the reconfiguration of organisational models grounded in scientific evidence [1,2]. In this context, comprehensive research into dietary behaviours, eating disorders (EDs), and body perception across the general population—spanning from adolescents and university students to elite athletes—represents a high-priority clinical and public health challenge [3,4,5,6,7]. Comprehending this phenomenon holistically requires a multifactorial analysis examining how sociodemographic variables, psychological factors—including low self-esteem and psychosocial factors evaluated during adolescence [5,8]—and sociocultural determinants simultaneously modulate individuals’ relationship with their nutrition and their own bodies [9,10,11].

The joint aetiopathogenesis of body image disturbances and disordered eating behaviours does not respond to an isolated causal model, but rather to the complex interaction between body dissatisfaction, distorted weight perception, and the internalisation of aesthetic ideals, such as the thin ideal and the athletic ideal [8,9,12]. Evidence provided by various studies employing specific assessment tools—such as the SATAQ-4 scale to evaluate the internalisation of body ideals and sociocultural pressures, and questionnaires such as the EAT-26 and EDE-Q for eating pathology screening—demonstrates that pressure exerted by the media, peers, and the family environment acts as a decisive catalyst in both the genesis of body dissatisfaction and the alteration in nutritional habits [8,9,10,13]. Likewise, prolonged exposure to digital environments and the problematic use of technologies are directly related to the internalisation of unrealistic aesthetic standards, the deterioration of diet quality, and an increase in pathological risks [1,13,14,15]. Nevertheless, research regarding the measurement invariance of constructs such as the SATAQ-4 warns of the need to interpret these assessments with caution when comparing diverse cultural or racial contexts [16], and highlights that media literacy interventions alone do not always successfully provide automatic protection for body image without a contextualised clinical approach [17].

Faced with this multifaceted reality, primary healthcare professionals and nursing teams face the urgent need to redesign their screening, prevention, and person-centred health education protocols to improve health literacy [2,17]. By fostering such innovations in clinical nursing practice and primary care systems, the combined use of dietary risk assessment instruments (such as the EAT-26 and EDE-Q) and scales assessing body perception and aesthetic pressure (such as the SATAQ-4) enables the identification of differential symptom profiles according to sex, BMI, and sociodemographic background [18,19,20]. Consequently, the systematic extraction of epidemiological and psychosocial data is essential for configuring e-Health interventions and educational programmes within primary care that act effectively and in parallel upon both body image mediators and nutritional habits [2,17,21,22]. Furthermore, when examining the impact of digital environments, it is crucial to move beyond mere screen-time duration and focus on specific psychological processes—such as online appearance comparison, exposure to idealized imagery, and feedback dynamics—which recent experimental and meta-analytic evidence identifies as critical drivers of body dissatisfaction and disordered eating symptoms [23,24].

The main purpose of this study is to investigate dietary behaviours and body perception in the general population according to various social and demographic variables, aiming to identify the profile of citizens who are most vulnerable and sensitive to eating disorders and body image alterations based on these sociodemographic profiles. This work seeks to provide managers and professionals with rigorous empirical information for the design of health policies and primary care health programmes that are person-centred and adapted to these risk profiles. Thus, this study aspires to drive advanced clinical practice and a more integrated, preventive public health model tailored to the real needs of users [1,2]. Ultimately, this study contributes to the core scope of this Special Issue by providing actionable, evidence-based foundations to advance nursing practice, shape preventive healthcare systems, and inform tailored, person-centred interventions aligned with the real needs of users.

## 2. Materials and Methods

### 2.1. Study Design

A quantitative, observational, descriptive, and cross-sectional study was carried out, with an analytical and associative design aimed at examining sociodemographic and behavioural variables in relation to the risk of eating disorders and sociocultural perception of body image.

### 2.2. Population and Sample

The target population consisted of the general public. Participants were selected through non-probability convenience sampling, yielding a final sample of 1287 participants. Due to the predominance of female participants, university students, and individuals with higher education levels, the sample is not statistically representative of the general population, and findings should be interpreted within this specific demographic context. Detailed sociodemographic and descriptive characteristics of the sample are presented in Table 1.

### 2.3. Data Collection and Instruments

Data were collected between 10 March and 1 April 2022 using a self-administered online questionnaire implemented on the official survey platform of the University of Murcia (https://encuestas.um.es/). Recruitment was facilitated through social media and the institutional invitation system.

The questionnaire comprised a sociodemographic section and two primary validated scales, widely described in the scientific literature:

- Eating Attitudes Test (EAT-26): A 26-item scale assessed on a Likert-type format (from “Never” to “Always”), which evaluates the risk of presenting an eating disorder. Scores range from 0 to 78, with the clinical risk cut-off established at ≥20 points. It is essential to emphasize that the EAT-26 functions strictly as a screening tool rather than a definitive diagnostic instrument. It is structured into three classical subscales according to its original validation (Garner et al. [25]): Dieting, Bulimia and Food Preoccupation, and Oral Control, utilizing the standardized Spanish-adapted version widely applied in research within this population. In the present sample, the scale demonstrated adequate internal consistency with a Cronbach’s alpha of 0.788.
- Sociocultural Attitudes Towards Appearance Questionnaire-4 (SATAQ-4): A 22-item scale on a Likert-type format (1 to 5) evaluating sociocultural influences on body image. Its global score ranges from 22 to 110, consisting of five subscales: Athletic Ideal Internalisation, Thin Ideal Internalisation, Family Pressure, Peer Pressure, and Media Pressure, implemented through its established Spanish-language adaptation. In this study, the SATAQ-4 showed acceptable internal consistency, with a Cronbach’s alpha of 0.716.

### 2.4. Variables

- Dependent variables: Global scores and subdomains of the EAT-26 and SATAQ-4 scales.
- Independent variables (and covariates):
  ○ Dichotomous/binomial variables: Sex (male/female), nationality (Spanish/foreign), residence area (urban/rural), smoking habit (yes/no), lifestyle (sedentary: <30 min of regular exercise less than 3 days per week, a threshold aligned with international public health standards to define insufficient physical activity and heightened health risks [26] without implying behavioral addiction, exercise dependence, or dietary hypervigilance), and level of internet and social media usage (adequate: ≤2 h/day; excessive: >2 h/day, a quantitative threshold established in alignment with screen-time exposure limits to prevent psychological distress [1] rather than indicating digital addiction, techno-stress, or clinical hyperconnectivity).
  ○ Multinomial variables: Marital status (single, married/cohabiting, divorced, widowed), commensality or eating habit (always accompanied, sometimes accompanied, never accompanied, classified strictly as a behavioral and relational frequency parameter without automatically assuming maladaptive coping strategies for emotional distress, as underlying motivations such as work schedules, living arrangements, or personal preferences require cautious individual assessment), and employment status (student, unemployed, employed, retired).
  ○ Continuous quantitative variables: Age and number of children.

### 2.5. Statistical Analysis

Normality of the distribution of quantitative variables was assessed using the Kolmogorov–Smirnov test. Descriptive analysis was conducted using frequencies and percentages for qualitative variables, and means, standard deviations, and ranges for quantitative variables. Bivariate analysis was performed using Student’s *t*-test for independent samples (for dichotomous contrasts) and one-way ANOVA (for multinomial variables), as well as Pearson’s correlation coefficient for continuous quantitative variables. Effect sizes were calculated using Cohen’s d and partial eta-squared. For multivariate analysis, multiple linear regression models were developed to identify variables independently associated with EAT-26 and SATAQ-4 scores. These models exclusively included independent variables that had demonstrated statistical significance in the prior bivariate analysis—a standard screening approach in exploratory cross-sectional designs aimed at preventing model overfitting and multicollinearity caused by sample saturation, while maintaining statistical control over core structural confounders. Polytomous categorical variables with multiple categories were modeled using dummy (indicator) variables with explicitly defined reference categories (commensality, marital status, and employment status). Additionally, a complementary binary logistic regression analysis was performed using the clinical cut-off point of EAT-26 ≥20 as the binary dependent variable, maintaining the same set of independent predictors to evaluate odds ratios (Exp(B)) and assess the robustness of the linear findings against a clinical risk threshold. Linear regression assumptions were comprehensively verified: multicollinearity was evaluated using the Variance Inflation Factor (VIF), normality of residuals through histograms and P-P plots, linearity and homoscedasticity via standardized residual scatterplots (ZRESID vs. ZPRED), and influential observations through Cook’s distance. For the binary logistic model, goodness-of-fit was evaluated using the Omnibus test of model coefficients and the Hosmer–Lemeshow test. Statistical significance was set at *p* < 0.05. Data processing was performed using IBM SPSS Statistics, version 26.0.

### 2.6. Ethical Considerations

The study was conducted in accordance with the ethical principles of the Declaration of Helsinki and strictly complied with current data protection regulations (Organic Law 3/2018). All participants were informed about the nature of the research, ensuring confidentiality, anonymity, and voluntary participation through a digital informed consent integrated at the beginning of the questionnaire. Following consultation with the relevant institutional body of the University of Murcia specifically through its research ethics advisory channels, the study was exempt from formal review by an ethics research committee as it was a voluntary, non-invasive opinion survey involving no direct intervention on subjects, and no formal protocol reference number is issued by the institution for such exempt, non-interventional studies.

## 3. Results

### 3.1. Descriptive Results

Regarding the lifestyle and consumption habits analysed in the total sample (N = 1287), eating sometimes accompanied predominated (55.8%), alongside an active lifestyle (57.8%) and a low prevalence of smoking (13.1%). Notably, more than half of the sample (64.3%) exhibited excessive internet and social media use (>2 h per day). Concerning questionnaire scores, the global mean for the EAT-26 was 9.86 (SD = 10.78), an overall mean value below the clinical cut-off point established at 20 points for eating disorder risk. Given that the EAT-26 functions strictly as a screening tool rather than a diagnostic instrument, it is noteworthy that 16% of the participants (*n* = 206) scored equal to or greater than the clinical cut-off point (≥20), thus indicating a positive screening for potential disordered eating risk. Regarding the SATAQ-4 questionnaire, the global mean score was 56.28 (SD = 16.24). Complete descriptive statistics, including ranges and reference clinical cut-offs for both scales and their subdomains, are detailed in Table 2.

### 3.2. Bivariate Analysis

Regarding the EAT-26 scale (Table 3), statistically significant differences were observed according to sex (M = 7.43, SD = 7.67 in males; M = 10.78, SD = 11.63 in females; *p* < 0.001, d = 0.33), smoking habit (M = 12.02, SD = 12.42 in smokers; M = 9.53, SD = 10.48 in non-smokers; *p* = 0.01, d = 0.27), lifestyle (M = 8.98, SD = 10.05 in sedentary; M = 10.50, SD = 11.25 in active; *p* = 0.01, d = 0.19), social media use (M = 8.30, SD = 9.29 in adequate use; M = 10.72, SD = 11.44 in excessive use; *p* < 0.001, d = 0.20), commensality (M = 8.50, SD = 9.36 always accompanied; M = 9.93, SD = 10.62 sometimes; M = 14.30, SD = 14.67 never; *p* < 0.001, η2= 0.022), and employment status (M = 10.59, SD = 11.58 in students; M = 9.33, SD = 8.86 unemployed; M = 8.69, SD = 9.40 active; M = 9.83, SD = 6.85 retired; *p* = 0.04, η2= 0.007). Conversely, no significant differences were found based on nationality, setting, marital status, or education level (*p* > 0.05). Regarding quantitative variables, age and number of children maintained weak but significant inverse correlations with the global EAT-26 score (r = −0.103, *p* < 0.001 and r = −0.067, *p* = 0.02, respectively).

Concerning the SATAQ-4 scale (Table 3), the analysis revealed significant differences based on sex (M = 52.83, SD = 15.33 in males; M = 57.83, SD = 16.39 in females; *p* < 0.001, *d* = 0.32), smoking habit (M = 58.77, SD = 17.38 in smokers; M = 55.90, SD = 16.03 in non-smokers; *p* = 0.03, d = 0.18), social media use (M = 51.88, SD = 15.08 in adequate use; M = 58.72, SD = 16.35 in excessive use; *p* < 0.001, d = 0.45), commensality (M = 53.77, SD = 15.33 always accompanied; M = 57.16, SD = 16.23 sometimes; M = 59.39, SD = 18.27 never; *p* < 0.001, η^2 = 0.014), marital status (M = 57.68, SD = 16.15 single; M = 55.03, SD = 16.11 married/partner; M = 46.45, SD = 15.35 divorced; M = 54.00, SD = 17.81 widowed; *p* < 0.001), and employment status (M = 57.93, SD = 15.93 in students; M = 61.08, SD = 18.50 unemployed; M = 53.45, SD = 16.54 active; M = 47.83, SD = 17.21 retired; *p* < 0.001, η^2 = 0.020). On the other hand, lifestyle, nationality, setting, and education level showed no globally significant differences (*p* > 0.05). Finally, age (r = −0.225, *p* < 0.001) and number of children (r = −0.173, *p* < 0.001) negatively and significantly correlated with perceived global sociocultural pressure.

### 3.3. Multivariate Analysis

The multiple linear regression models identified the predictor factors maintaining independent statistical association (Table 4), showing overall model fit statistics of $R^{2}=0.068$ (adjusted $R^{2}=0.060$, $F\left(11,1274\right)=8.450$, p<0.001) for the EAT-26 model, and $R^{2}=0.101$ (adjusted $R^{2}=0.092$, $F\left(13,1273\right)=10.981$, p<0.001) for the SATAQ-4 model.

Regarding the EAT-26, higher scores were observed in association with female sex (B=3.23; 95% CI $\left[1.92,\;\;4.55\right]$; β=0.134; p<0.001), active individuals (B=2.04; 95% CI $\left[0.87,\;\;3.21\right]$; β=0.094; p=0.001), smokers (B=2.89; 95% CI $\left[1.18,\;4.61\right]$; β=0.091; p=0.001), excessive social media use (B=1.98; 95% CI $\left[0.71,\;3.25\right]$; β=0.088; p=0.002), and specifically never eating in company compared to always (Commensality: Never vs. Always, B=5.59; 95% CI $\left[3.47,\;7.71\right]$; β=0.149; p<0.001); whereas eating sometimes (vs. always) did not reach statistical significance (p=0.107).

Regarding the SATAQ-4 scale, greater susceptibility to the influence of sociocultural factors on body image was found in females (B=3.43; 95% CI $\left[1.48,\;5.38\right]$; β=0.094; p=0.001), those who smoked (B=3.76; 95% CI $\left[1.22,\;6.30\right]$; β=0.078; p=0.004), those with excessive social media use (B=5.39; 95% CI $\left[3.50,\;7.27\right]$; β=0.159; p<0.001), younger age (B=−0.24; 95% CI $\left[-0.37,-0.12\right]$; β=−0.202; p<0.001), and both categories of commensality relative to always eating in company—specifically sometimes (B=2.53; 95% CI $\left[0.66,\;4.41\right]$; β=0.078; p=0.008) and never (B=4.53; 95% CI $\left[1.37,\;7.69\right]$; β=0.082; p=0.005). Following multivariate adjustment, categorical variables modeled via dummy variables (employment status and marital status categories) as well as age and number of children showed no statistically significant association in the EAT-26 model; analogously, employment status categories, marital status categories, and number of children did not present significant associations in the SATAQ-4 model.

### 3.4. Binary Logistic Regression Analysis

The binary logistic regression model identified the predictor factors maintaining independent statistical association with a high risk of eating disorders (Table 5), showing overall model fit statistics of Nagelkerke $R^{2}=0.100$, Omnibus test $\chi^{2}\left(11\right)=77.951$, p<0.001, and a good model fit according to the Hosmer–Lemeshow test ($\chi^{2}\left(8\right)=11.234$, p=0.189).

The probability of scoring at or above the clinical cutoff on the EAT-26 was associated with female sex (B=0.978; 95% CI $\left[1.728,\;4.093\right]$; $\mathrm{Exp}\left(B\right)=2.660$; p<0.001), active individuals (B=0.406; 95% CI $\left[1.088,\;2.070\right]$; $\mathrm{Exp}\left(B\right)=1.500$; p=0.013), smokers (B=0.868; 95% CI $\left[1.580,\;3.601\right]$; $\mathrm{Exp}\left(B\right)=2.381$; p<0.001), excessive social media use (B=0.510; 95% CI $\left[1.154,\;2.404\right]$; $\mathrm{Exp}\left(B\right)=1.665$; p=0.006), and specifically never eating in company compared to always (Commensality: Never vs. Always, B=0.812; 95% CI $\left[1.338,\;3.792\right]$; $\mathrm{Exp}\left(B\right)=2.253$; p=0.002); whereas eating sometimes (vs. always) did not reach statistical significance (p=0.301). Furthermore, age showed a slight inverse association (B=−0.031; 95% CI $\left[0.945,\;0.995\right]$; $\mathrm{Exp}\left(B\right)=0.969$; p=0.019), while a greater number of children was associated with an increased likelihood of risk (B=0.338; 95% CI $\left[1.043,\;1.884\right]$; $\mathrm{Exp}\left(B\right)=1.402$; p=0.025). Following multivariate adjustment, categorical variables modeled via dummy variables (employment status categories) showed no statistically significant association.

## 4. Discussion

The primary purpose of this study was to investigate dietary behaviors and the perception of corporeality within the general population to identify risk profiles and vulnerabilities regarding eating disorders (EDs) and body image alterations. Although the overall mean score on the EAT-26 scale fell below the clinical cutoff point, the multivariate analysis validates and precisely operationalizes our initial hypotheses by delineating a distinct multifactorial vulnerability profile.

On the one hand, the variables independently associated with higher scores indicating an increased risk of altered eating behaviors (EAT-26) comprise female sex, an active lifestyle (likely associated with a heightened focus or obsession toward weight control and dietary regulation), tobacco use (frequently explained by smoking as a dysfunctional weight-control mechanism), excessive social media use, and solitary eating habits (specifically never eating in company compared to always). On the other hand, the profile exhibiting heightened susceptibility to sociocultural pressures regarding body image (SATAQ-4) is specifically concentrated among young women, smokers, hyperconnected individuals, and those who eat alone, whether occasionally or never (commensality). These findings demonstrate that risk is not driven by isolated factors but rather by the convergence of behavioral, digital, and relational variables that require comprehensive management (see Figure 1).

When contrasting our results with preceding literature, a strong consonance is observed with studies emphasizing the multicausal nature of body image alterations and disordered eating habits. First, the heightened vulnerability detected in females (across both the EAT-26 and the SATAQ-4) aligns robustly with the international literature—such as the works by Piko et al. [3], Sahlan et al. [11], and Alkazemi et al. [19]—which documents that women experience intensified sociocultural pressure driven by the internalization of thinness ideals. Nevertheless, our data also reaffirm the necessity of addressing gender nuances and the pressure exerted by emerging body ideals described in alternative works.

The finding that an active lifestyle constitutes a correlate of risk for eating behaviors directly dialogues with prior investigations involving university populations, elite athletes, and adolescents, such as those by Alpay et al. [4], Torres-McGehee et al. [6], Dobkowska-Szefer et al. [7], and Harrison et al. [22]. These studies caution against the fine line existing between healthy exercise practices and the development of pathological weight-control behaviors, exercise addiction, or dietary hypervigilance.

In addition, the binary logistic findings underscore the complementary predictive role of specific sociodemographic factors such as age and family structure (number of children), pointing to life-stage transitions as critical windows of vulnerability that warrant careful consideration in preventive frameworks [5].

Likewise, the strong predictive capacity of excessive social media use robustly supports recent studies by Chaput and LeBlanc [1], Blackburn and Hogg [13], Aydin Cil et al. [14], Prybutok et al. [15] and Seslikaya and Arslan [27], which indicate that prolonged exposure to digital environments and unrealistic aesthetic contents is closely associated with nutritional deterioration, problematic internet use, and body dissatisfaction. Furthermore, as pointed out by various investigations on media literacy and embodied resilience, such as that by Schaben et al. [17], technological use increases pathology when it is not accompanied by critical resilience and an appropriate contextualized clinical approach.

Finally, the relevance solitary eating (non-commensality, specifically never eating with others, as well as occasional commensality in the case of sociocultural body pressure) and smoking habits as independent predictors complements the literature on social determinants and behavioral correlates. This evidences that meal isolation and substance consumption act as markers of maladaptive coping behaviors against bodily and emotional distress, in close accordance with the links between social interaction avoidance and sociocultural pressure described by authors such as Azevedo and Azevedo [10].

The findings of this study yield empirical information of substantial value that can help generate hypotheses for targeted screening and future interventions. Translating these results into real-world practice ultimately demands that longitudinal validation and intervention studies be conducted before formulating definitive clinical or health policy recommendations. Given that tools such as the EAT-26 and the SATAQ-4 enable the identification of vulnerabilities before severe clinical disorders manifest, nursing professionals may one day systematically integrate inquiries regarding social media exposure duration, commensality habits, and active lifestyle contexts into their comprehensive assessments, once validated by further research.

Having a clear risk profile (observed among young women, smokers, hyperconnected individuals, and those with meal-isolation habits) facilitates the design of potential primary and secondary prevention strategies. Moreover, these data support the future implementation of educational programs grounded in digital health literacy and the promotion of e-Health interventions from the primary care level, thereby optimizing preventive resources and fostering a more anticipatory, person-centered public health model.

Despite the robustness and clinical relevance of the obtained results, data must be interpreted in light of certain methodological limitations that present opportunities for improvement in future research. Firstly, the cross-sectional design of the study precludes establishing definitive causal relationships between behavioral variables (such as social media use or commensality) and the development of at-risk eating behaviors, capturing a single temporal snapshot of the phenomenon. Secondly, the use of non-probabilistic convenience sampling—characterized by a high proportion of female participants, university students, and individuals with higher education levels—strictly limits the generalization of our findings to the broader general population. Moreover, this non-probabilistic sampling involved partial recruitment through social media platforms, which introduces a potential selection bias by preferentially capturing individuals with inherently higher levels of digital interaction. Thirdly, because data were collected between March and April of 2022, the rapidly evolving nature of social media platforms and digital behavior means that these findings on digital exposure might not fully reflect current usage patterns. In addition, the evaluation of digital media is subject to several constraints: the operationalization relied on a simplistic dichotomization of exposure time, lacked the ability to distinguish between passive versus active usage or appearance-focused interactions, and did not capture qualitative processes such as appearance comparisons. Furthermore, the study did not include Body Mass Index (BMI) in the final multivariate regression models despite its potential confounding role, and the possibility of residual confounding due to unmeasured psychosocial variables cannot be ruled out. Finally, the use of self-reported online questionnaires may be subject to social desirability or self-reporting biases in estimating behavioral and symptomatological variables. Far from detracting from rigor, these methodological limitations are interpreted as areas of opportunity to consolidate future longitudinal designs.

As a natural continuation of this work, several future research avenues are proposed to overcome the outlined limitations and deepen the understanding of the phenomenon. Primarily, prioritizing the development of longitudinal and quasi-experimental intervention studies that evaluate the efficacy of prevention programs based on health technologies (e-Health) and media literacy in primary care is essential to validate these findings prior to any definitive clinical implementation. Secondly, incorporating complementary objective measures and expanding the analysis to direct metabolic and nutritional variables would be of significant interest, alongside conducting cross-cultural comparative studies examining the invariance of body image constructs across diverse sociodemographic contexts. Finally, further exploring the nature of active lifestyles and their specific interaction with mental health and body image across different segments of the general population will constitute a key avenue for refining clinical practice guidelines within the primary care setting.

## 5. Conclusions

Based on this adult population sample, the findings demonstrate that vulnerability to eating disorders and body image alterations is defined by a distinct multifactorial profile. This risk profile is prominently concentrated among women, smokers, solitary eaters, digitally hyperconnected individuals, and those with an active lifestyle, a pattern robustly confirmed by the binary logistic sensitivity analysis identifying clinical risk thresholds (EAT-26 ≥ 20).

These results establish that altered eating behaviors and sociocultural pressures are driven by the convergence of behavioral, relational, and technological variables, alongside demographic factors such as younger age and family size. Consequently, these descriptive and associative insights provide a solid empirical foundation to guide targeted clinical screening and person-centered nursing interventions within healthcare systems.

## Figures and Tables

**Figure 1:**
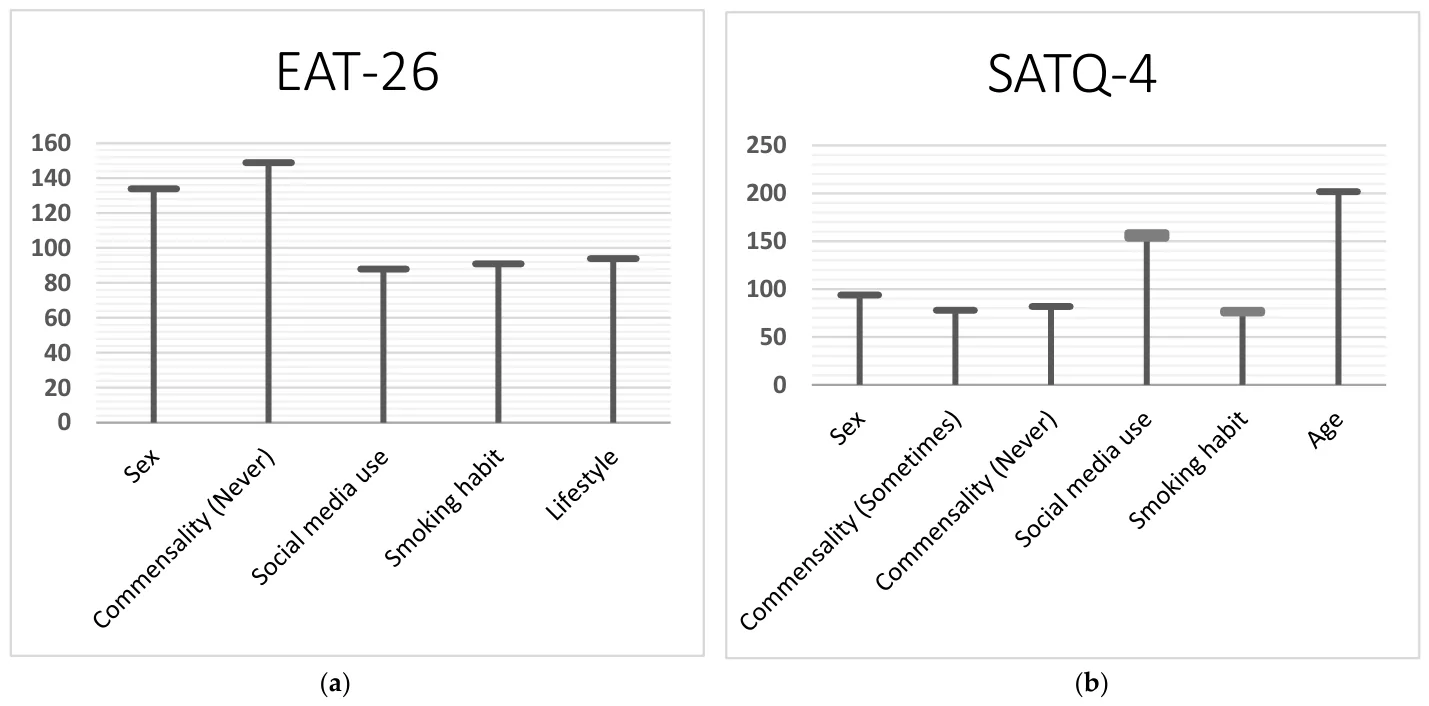
Multifactorial risk profiles based on standardized regression coefficients (β). (**a**) Predictors of disordered eating risk (EAT-26); (**b**) predictors of sociocultural body image pressure (SATAQ-4). Note: Only statistically significant variables (*p* < 0.05) are shown. Absolute values of coefficients are displayed for comparative visual layout, with age reflecting an inverse (negative) association (β = −0.202).

**Table 1:** Description of the sample.

Variables	Categories	*n*	%
Sex	Male	355	27.6
	Female	932	72.4
Nationality	Spanish	1213	94.3
	Foreign	74	5.7
Marital status	Single	714	55.5
	Married	532	41.3
	Divorced	33	2.6
	Widowed	8	0.6
Education level	Primary	6	0.5
	Secondary	23	1.8
	High School	275	21.4
	Higher Education	983	76.4
Employment status	Student	778	60.5
	Unemployed	24	1.9
	Active	479	37.2
	Retired	6	0.5
Setting	Urban	1080	83.9
	Rural	207	16.1
Quantitative variables		**X**	**SD**
Age		29.87	13.12
Number of children		0.42	0.90

Note: n = Absolute frequency; % = Percentage; SD = Standard Deviation.

**Table 2:** Descriptive statistics of behavioural variables and scores on the EAT-26 and SATAQ-4 scales.

**Variables**	**Categories**	***n***	**%**
Commensality	Always	445	34.6
	Sometimes	718	55.8
	Never	124	9.6
Lifestyle	Sedentary	543	42.2
	Active	744	57.8
Smoking habit	Yes	169	13.1
	No	1118	86.9
Social media use	Adequate	459	35.7
	Excessive	828	64.3
**Scale**	**Subdomains**	**Mean (SD)**	**Range**
EAT-26 (Global)	Total Score	9.86 (10.78)	75
	Dieting Subdomain	3.10 (4.01)	24
	Bulimia and Food Preoccupation	5.39 (6.71)	36
	Oral Control Subdomain	1.35 (2.17)	15
SATAQ-4 (Global)	Total Score	56.28 (16.24)	84
	Muscle Internalisation	13.33 (4.84)	20
	Thinness Internalisation	14.93 (5.23)	20
	Family Pressure	8.65 (4.36)	16
	Peer Pressure	6.86 (3.57)	16
	Media Pressure	12.50 (5.49)	16

Note: *n* = Absolute frequency; % = Percentage; SD = Standard Deviation.

**Table 3:** Bivariate analysis of independent variables in relation to the global EAT-26 and SATAQ-4 scores.

Variables	Categories	EAT-26 Total			SATAQ-4 Total		
		M (SD)	*p*	d/η2	M (SD)	*p*	d/η2
Sex	Male	7.43 (7.67)	<0.001	0.33	52.83 (15.33)	<0.001	0.32
	Female	10.78 (11.63)			57.83 (16.39)		
Nationality	Spanish	8.88 (10.67)	0.13	-	56.20 (16.22)	0.49	-
	Foreign	11.68 (12.43)			57.53 (16.57)		
Setting	Urban	9.82 (10.73)	0.75	-	56.60 (16.13)	0.10	-
	Rural	10.08 (11.06)			54.58 (16.72)		
Lifestyle	Sedentary	8.98 (10.05)	0.01	0.19	55.91 (16.39)	0.48	-
	Active	10.50 (11.25)			56.55 (16.14)		
Smoking habit	No	9.53 (10.48)	0.01	0.273	55.90 (16.03)	0.03	0.18
	Yes	12.02 (12.42)			58.77 (17.38)		
Social media use	Adequate	8.30 (9.29)	<0.001	0.20	51.88 (15.08)	<0.001	0.45
	Excessive	10.72 (11.44)			58.72 (16.35)		
Commensality	Always	8.50 (9.36)	<0.001	0.02	53.77 (15.33)	<0.001	0.01
	Sometimes	9.93 (10.62)			57.16 (16.23)		
	Never	14.30 (14.67)			59.39 (18.27)		
Marital status	Single	10.26 (11.37)	0.38	-	57.68 (16.15)	<0.001	0.016
	Married	9.26 (9.99)			55.03 (16.11)		
	Divorced	9.73 (8.90)			46.45 (15.35)		
	Widowed	13.75 (14.18)			54.00 (17.81)		
Education level	Primary	8.67 (10.13)	0.93	-	52.00 (15.00)	0.59	-
	Secondary	10.39 (10.53)			56.61 (14.64)		
	High School	10.19 (12.07)			57.35 (16.57)		
	Higher Education	9.76 (10.42)			56.00 (16.20)		
Employment status	Student	10.59 (11.58)	0.04	<0.001	57.93 (15.93)	<0.001	0.020
	Unemployed	9.33 (8.86)			61.08 (18.50)		
	Active	8.69 (9.40)			53.45 (16.54)		
	Retired	9.83 (6.85)			47.83 (17.21)		
Age		r = −0.103	<0.001		r = −0.225	<0.001	
Number of children		r = −0.067	0.02		r = −0.173	<0.001	

Note: M = Mean; SD = Standard Deviation; *p* = Statistical significance; d = Cohen’s d; η2 = Partial Eta squared; r = Pearson’s correlation coefficient.

**Table 4:** Multiple linear regression for EAT-26 and SATAQ-4.

Variables	EAT-26				SATAQ-4			
	B [CI 95%]	β	*p*	VIF	B [CI 95%]	β	*p*	VIF
(Constant)	3.43 [−2.51 to 9.38]	-	0.257	-	54.38 [45.95 to 62.82]		<0.001	
Sex	3.23 [1.92 to 4.55]	0.134	<0.001	1.058	3.43 [1.48 to 5.38]	0.094	0.001	1.060
Lifestyle	2.04 [0.87 to 3.21]	0.094	0.001	1.024	-			
Smoking habit	2.89 [1.18 to 4.61]	0.091	0.001	1.027	3.76 [1.22 to 6.30]	0.078	0.004	1.025
Social media use	1.98 [0.71 to 3.25]	0.088	0.002	1.130	5.39 [3.50 to 7.27]	0.159	<0.001	1.136
Commensality (Ref.: Always)								
Sometimes vs. Always	1.03 [−0.22 to 2.29]	0.048	0.107	1.201	2.53 [0.66 to 4.41]	0.078	0.008	1.213
Never vs. Always	5.59 [3.47 to 7.71]	0.149	<0.001	1.197	4.53 [1.37 to 7.69]	0.082	0.005	1.213
Employment status (Ref.: Active)								
Student vs. Active	0.42 [−1.22 to 2.107]	0.019	0.615	1.981	−1.56 [−4.00 to 0.88]	−0.047	0.210	1.994
Unemployed vs. Active	0.24 [−4.07 to 4.57]	0.003	0.910	1.049	6.29 [−0.10 to 12.69]	−0.052	0.054	1.046
Retired vs. Active	−0.17 [−8.85 to 8.50]	−0.001	0.968	1.069	−1.07 [−15.04 to 12.89]	−0.005	0.880	1.265
Marital status (Ref.: Single)								
Married vs. Single	-				1.34 [−0.69 to 3.38]	0.041	0.197	1.409
Divorced vs. Single	-				−5.60 [−11.45 to 0.24]	−0.055	0.060	1.193
Widowed vs. Single	-				6.97 [−5.40 to 19.36]	0.034	0.269	1.322
Age	−0.06 [−0.14 to 0.18]	−0.079	0.124	3.654	−0.24 [−0.37 to −0.12]	−0.202	<0.001	3.771
Number of children	0.81 [−0.18 to 1.81]	0.068	0.109	2.489	0.30 [−1.20 to 1.81]	0.017	0.695	2.601

Model summary statistics: For the EAT-26 model: R^2^ = 0.068, Adjusted R^2^ = 0.060, F(11,1274) = 8.450, *p* ≪ 0.001. For the SATAQ-4 model: R^2^ = 0.101, Adjusted R^2^ = 0.092, F(13,1273) = 10.981, *p* ≪ 0.001. Abbreviations: B = Unstandardized regression coefficient; CI 95% = 95% Confidence Interval; β = Standardized coefficient; *p* = Statistical significance; VIF = Variance Inflation Factor. Methodological Notes: Categorical variables with multiple categories were modeled using dummy (indicator) variables with explicitly defined reference categories: Commensality (Reference: Always accompanied), Marital status (Reference: Single), and Employment status (Reference: Active/Employed). Binary variables were coded as dummy indicators (Sex: 0 = Male, 1 = Female; Lifestyle: 0 = Sedentary, 1 = Active; Smoking habit: 0 = No, 1 = Yes; Social media use: 0 = Adequate, 1 = Excessive).

**Table 5:** Results of the binary logistic regression model for the binary EAT-26.

Variable/Predictor	B	S.E.	Wald	df	*p*	Exp(B)	95% CI
(Constant)	−2.81	0.851	10.905	1	0.001	0.060	-
Sex	0.978	0.220	19.770	1	<0.001	2.660	[1.728 to 4.093]
Lifestyle	0.406	0.164	6.113	1	0.013	1.500	[1.088 to 2.070]
Smoking habit	0.868	0.209	17.196	1	<0.001	2.381	[1.580 to 3.601]
Social media use	0.510	0.187	7.422	1	0.006	1.665	[1.154 to 2.404]
Commensality (Ref.: Always)							
Sometimes vs. Always	0.186	0.180	1.069	1	0.301	1.205	[0.846 to 1.715]
Never vs. Always	0.812	0.266	9.340	1	0.002	2.253	[1.338 to 3.792]
Employment status (Ref.: Active)							
Student vs. Active	0.089	0.233	0.146	1	0.702	1.093	[0.692 to 1.724]
Unemployed vs. Active	−0.521	0.770	0.457	1	0.499	0.594	[0.131 to 2.687]
Retired vs. Active	−0.123	1.312	0.009	1	0.925	0.884	[0.068 to 11.569]
Age	−0.031	0.013	5.540	1	0.019	0.969	[0.945 to 0.995]
Number of children	0.338	0.151	5.023	1	0.025	1.402	[1.043 to 1.884]

For the binary logistic model: Nagelkerke R^2^ = 0.100, Omnibus test X^2^ (11) = 77.951, *p* < 0.001, Hosmer–Lemeshow test X^2^ (8) = 11.234, *p* = 0.189. Abbreviations: B = Unstandardized regression coefficient (Log-Odds); S.E. = Standard Error; Wald = Wald statistic; df = Degrees of freedom; *p* = Statistical significance; Exp(B) [OR] = Odds Ratio; 95% CI = 95% Confidence Interval for Exp(B).

## Data Availability

The data supporting the findings of this study are available from the corresponding author upon reasonable request. The data are not publicly available due to privacy and ethical restrictions.

## Abbreviations

The following abbreviations are used in this manuscript:

EDs	Eating Disorders
SATAQ-4	Sociocultural Attitudes Towards Appearance Questionnaire-4
EAT-26	Eating Attitudes Test-26
EDE-Q	Eating Disorder Examination Questionnaire
BMI	Body Mass Index
